# Comparative study of neonatal hypothermia and associated factors among neonates in rural and urban areas of the Shebadino Woreda, Sidama region, Southern Ethiopia: a community-based comparative cross-sectional study

**DOI:** 10.1186/s12889-024-19504-8

**Published:** 2024-07-20

**Authors:** Gizu Tola Feyisa, Shambel Negese Marami, Dagne Deresa Dinagde, Bekem Dibaba Degefe, Shimelis Tadesse Abebe, Gemeda Wakgari Kitil, Andargachew Kassa Biratu

**Affiliations:** 1 Department of Midwifery, College of Health Sciences, Mattu University, Mettu, Ethiopia; 2 Department of Midwifery, College of Medicineand,, Health Sciences , Hawassa University, Hawassa, Ethiopia

**Keywords:** Community-based study, Hypothermic, Neonates, Non-hypothermic, Sidama, Ethiopia

## Abstract

**Background:**

Hypothermia is one of the major causes of newborn death, particularly in low-income nations. This was due to poor thermal care in most of the rural communities. Recent studies show that there was a prevalence discrepancy between urban and rural communities where economic, educational, and life standard differences exist. Therefore, this study aimed to assess the prevalence and factors associated with neonatal hypothermia among neonates in rural and urban areas of the Shebadino woreda, Sidama region, Ethiopia.

**Method:**

A comparative community-based cross-sectional study was performed on 682 neonates in the Shebadino Woreda, Sidama Region, southern Ethiopia, in 2023. A multistage sampling technique was used, and the collected data were manually cleaned, coded, and entered into Epi Data version 4.6 before being exported to SPSS version 26 software for analysis. Variables with a p-value < 0.25 in the bivariate logistic regression were further analyzed using multivariable logistic regression. The odds ratio (OR) with 95% CI was used as a measure of association, and variables that had a p-value less than 0.05 in the multivariable logistic regression were considered significantly associated variables.

**Results:**

The overall prevalence of neonatal hypothermia in this study was 51.8% (95% CI: 47.2%-56.3%). It was greater among rural neonates (55.1%) than among urban neonates (48.6%). Bathing before 24 h. (AOR = 3.64, 95% CI: 1.39, 7.16), Placing a cold object near babies’ head (AOR = 2.97, 95% CI: 1.75, 5.03), Neonates who were given traditional medication (Amessa) (AOR = 1.83% CI; 1.04–3.20) and, not separated humans and animals house (AOR = 1.75, 95%, 1.05–2.91) were significantly associated with neonatal hypothermia in rural, while Night time delivery (AOR = 1.81, CI: 1.01–5.62), Neonates who were given traditional medication (Amessa) (AOR = 3.11% CI; 1.85–5.21), and Placing a cold object near babies’ head (AOR = 2.40, 95% CI: 1.37, 3.29 were significantly associated with neonatal hypothermia among urban neonates.

**Conclusion:**

The Prevalence of neonatal hypothermia in the study area was relatively greater in rural areas than in urban areas. Cost-effective thermal care such as separating humans from animal houses, teaching not to put cold objects near babies, giving special care to newborns for those delivered from women with medical problems, and giving priority to those delivered at night, is needed.

**Supplementary Information:**

The online version contains supplementary material available at 10.1186/s12889-024-19504-8.

## Background

Neonatal hypothermia was defined by the World Health Organization (WHO) as a body temperature of 36.5 °C (97.7°F) or less before the age of 28 days. If the axillary temperature ranged from 36.0 °C to 36.5 °C, mild hypothermia occurred; if the temperature ranged from 32.0 °C to 36.0 °C, moderate hypothermia occurred; and if the temperature was 32.0 °C, severe hypothermia occurred. Newborns are vulnerable to environmental interference as they face life outside the womb due to their systemic immaturity [1]. Heat generation does not exceed heat loss as a newborn’s body temperature drops from 2 °C to 3 °C in the first half-hour of life [1, 2]. Heat can be lost through evaporation (due to the evaporation of amniotic fluid from the skin surface), conduction by contact with cold objects, such as clots), convection (by air currents in which cold air replaces warm air around baby-open windows), and radiation [1].

The warm delivery room, immediate drying, skin-to-skin contact, breastfeeding, postponing weighing and bathing, providing appropriate clothing and bedding, keeping mothers and babies together, warm transportation, warm resuscitation, training, and raising awareness are the ten interlinked warm chain preventive mechanisms recommended by the WHO [1]. In contrast, hypothermia is still a major problem in poor countries, particularly in sub-Saharan Africa [3]. Poverty, home delivery, low birth weight, early bathing, delayed commencement of breastfeeding, and limited understanding among health personnel are all risk factors for neonatal hypothermia in the region [4].

Hypothermia plays a significant role in neonatal deaths, with global case fatality rates (CFRs) ranging from 8.5% to 52% [2]. Mortality increased by approximately 80% for every degree Celsius of decrease in the first observed axillary temperature. The relative risk of death ranged from 2 to 30 times within the current WHO classification for moderate hypothermia, increasing with greater severity of hypothermia [3]. Approximately 20% of deaths are caused by prematurity; however, better thermal care could prevent 10% of mortality in term newborns [4]. Its presence in most morbidity cases is more evident than it is in other cases, as it is related to a peri-intraventricular hemorrhage grade 3 and death [5]. According to recent studies, the mortality rate increases fivefold when the temperature of newborns drops by one degree Celsius, and every one-degree decrease in body temperature raises mortality by 80 percent [6]. It is particularly common at home in low-income countries with weak health systems, where even health professionals have a poor understanding of hypothermia as a risk to the newborn [2].

In sub-Saharan Africa, many births and deaths occur at home, which worsens the problem of taking accurate vital records. In contrast, there is no similar community-based research in sub-Saharan Africa except one research conducted in the Lira district of Uganda. As a result, the actual incidence of newborn hypothermia in the region is unknown [7, 8]. Therefore, the neonatal mortality caused by hypothermia may exceed the estimated mortality based on complete data [3]. Additionally, the temperature is not taken, in most newborns, immediately after birth. The home delivery rate is high in the study area [9], and there was cultural malpractice applied to newborns at home in Shebadino Woreda south Ethiopia according to recent research. Additionally, the lack of transportation for moving from house to house during home visits by health extension workers, absence of all-weather roads for ambulance services in some rural kebeles, work overload for Health extension workers, budget shortages, absence of regular monitoring and evaluation of the program by the District Health Office and negligence by HEWs due to long time services without promotion and benefits are the major challenges in Community-based neonatal care (CBNC) in rural communities [10]. Recent research also showed that there is a prevalence discrepancy between neonates from rural and urban [11]. This implies that additional research should be performed using a better design and methodological sound of community-based studies to understand the problem more, and more. Therefore, the purpose of this study was to determine the prevalence and associated factors in rural and urban areas of the Shebadino Woreda Sidama region in southern Ethiopia. With the results of this study, NGOs working in this area will get important information that will help to address the gaps and run for solutions. This study has also a greater input for our country Ethiopia, and other developing countries to achieve the Sustainable Development Goal Three (SDG) targets, which was proposed to decrease neonatal mortality to 12 deaths per 1000 live births by 2030.

## Methods

### Study settings

The research was carried out in Shebadino woreda, a territory of the Sidama National Regional State. Its elevation ranges from 1750 to 3000 m above sea level. Shebadino woreda has an annual average rainfall of 1200 mm, with maximum and minimum values of 1600 mm and 800 mm, respectively. According to the Regional and Woreda metrology report, the average annual temperature is 28.5 °C, with hot days reaching a maximum of 31 °C in February and March and cold days reaching 21 °C and 22 °C in July and August, respectively. This region has 86 percent of the country’s midland area, 14 percent of its highland area, and nearly no lowland climate zones. According to previous studies, pneumonia is the most common health condition in the region with 33.5% [12].

### Study design and period

Data was collected from June 10 to November 10, 2022, with a community-based Comparative cross-sectional study.

### Population

The study population included neonates and their mothers from selected kebeles of Shebadino woreda during the actual data collection period**.** However, neonates’ mothers who stayed in the woreda for less than six months were excluded from the study. Preterm neonates and newborns whose mothers were suffering from critical illness (postpartum hemorrhage) or postpartum psychosis were also excluded.

### Sample size

The sample size was estimated using a double proportion formula, considering the proportion of patients with neonatal hypothermia (53% of whom 40% were urban) from a previous study conducted at Arbaminch General Hospital [11]. The formula and calculation are as follows:

N (in each group) = (p_1_q_1_ + p_2_q_2_) (f (α, β))/((p_1_—p_2_).^2^

where *n* = the sample size for each group.

P1 = proportion of neonatal hypothermia (rural 53%).

P2 = the proportion of neonatal hypothermia (40%).

F (α, β) = 7.84, power = 80% and significance = 5%

q1 = (1-p1) = 1–0.53 = 0.47.

q2 = (1-p2) = 1–0.4 = 0.6

n1 = n2 = (0.84 + 1.96)^2^ ((0.47 × 0.53) + (0.4 × 0.6))/(0.53 -0.4)^2^ = 227.

The total sample size for both groups = 454.

With a design effect of 1.5, i.e., 454*1.5 = 681, by adding a 5% nonresponse rate, the final estimated total sample size for this study was 716 (358 and 358 study subjects from rural and urban areas, respectively).

### Sampling procedure

There are 30 kebeles (the smallest administrative unit in the district), which means there are 25 rural kebeles and 5 urban kebeles. A simple random sampling procedure was used to select 40% (10 rural Kebeles) of all kebeles (the smallest administrative entities in an Ethiopian district). At six months, 2154 neonates were born to 10 rural kebeles, whereas 632 neonates were born to three urban kebeles from the previously reported data to health extension workers in each Kebeles. The determined sample size was proportionally allocated to the population size in each selected kebele. To identify study individuals, a multistage sampling procedure was used. Then, participants were selected by using a systematic random sampling technique in order of birth registration from the health extension worker; that is, every two birth reports until the required sample size was obtained for urban areas (K = 1.76; approximately every 2 neonate birth reports were taken), whereas the K value for rural areas was 6. Households of the women who gave birth were identified and reached with the help of the health extension worker and Health Development Army of those Selected Kebeles.

### Operational definitions

**Non-hypothermic:** an axillary neonatal temperature ≥ 36.5 °C at the time the data collector arrives at home [13].

**Hypothermic:** an axillary neonatal temperature < 36.5 °C at the time the data collector arrived at the hospital.

**Moderate hypothermia**: an axillary temperature of 32.0 to 35.9 °C.

**Severe hypothermia:** an axillary temperature of < 32.0 °C.

**Hyperthermia:** an axillary temperature of > 37.5 °C.

### Data collection tools and procedures

Six BSc midwives and two MSc supervisors were used for data collection and measurements from the mothers after home delivery. All the data collectors and supervisors were chosen based on their prior data collection experience and their proficiency in reading, writing, speaking, and understanding in Sidama affo and English. The data collector went into every home and gathered data after each birth with the aid of the HEW and the health development army. The data were collected for mothers who gave birth in a medical facility as soon as the mother was taken home.

The studies performed at Addis Abeba and Arbminch General Hospital were refined and utilized as the basis for a structured, interviewer-administered questionnaire [11, 14]. The interview questionnaire was written in English, translated into the regional Sidamo affo language, and then translated back into English to ensure uniformity.

The personnel responsible for supervising the data collection were trained to measure neonatal temperature, baby weight, and room temperature and to calculate gestational age. During the study visit, the temperature was measured using a digital thermometer at the axilla until an audible beep was heard automatically. These are the ones we picked since they are accessible locally, affordable, and simple for community workers to utilize. Additionally, we utilized axillary measurements rather than rectal measurements because they are simpler, safer, and more socially acceptable [15].

The axillary temperature of the newborn was measured as soon as home arrival by a digital thermometer (model- MT-101), which has a measurement accuracy of ± 0.1 °C for the temperature range from 35.5 °C–42.0 °C and ± 0.2 °C for the temperature range of 32.0 °C–35.5 °C or above 42.0 °C [14, 16]. The measurement was performed by placing a thermometer in the baby's armpit. With an emphasis on reducing the amount of time the babies may be exposed to the cold, the temperature of the baby was measured before the baby's weight was taken by a professional data collector. To ensure high reliability, two measurements in degrees Celsius were made repeatedly at the same time, and the average of these two values was used. The temperature measurement was cross-checked with reference thermometers every week to avoid any false readings due to possible damage to the thermometers during transportation. With 70% ethyl alcohol disinfectant the thermometer was disinfected with a damp cloth after every measure of axillary temperature of the newborn to prevent infection transmission.

The room temperature was also measured by a Gera Mercury thermometer with a range of − 37 °C to 356 °C and a measurement accuracy of ± 3 °C. As soon as we arrived home, we positioned the mercury thermometer more than 2 feet above the ground in the middle of the room and waited at least 15 min for the temperature to read. The gestational age was calculated based on the woman's account of her most recent period (Last normal menstrual period), and an early ultrasound record from the mother. Weight was measured using a weighing scale called the RGZ 20, which was accurate to within 50 g [14]. The oxygen saturation and pulse rates were determined using a pulse oximeter.

### Data quality assurance

In the Arbegona woredas, which had a setting similar to the study site, the pretest of the data collection was conducted on 36 neonates, or 5% of the sample size. This pretest was created to determine how long it would take, whether the answers were accurate, the language was clear, and whether the data collection tool was adequate. In-depth training covering the research objective, the list of eligible study participants, the tools and procedures for collecting data, and interviewing techniques were provided to the data collectors over two days. The principal investigator, data collectors, and supervisors verified the accuracy of the data each day before entering it. Additionally, each questionnaire was crosschecked with the entered data, and all observed errors were corrected. The thermometers were calibrated’ (the measurement was crosschecked with reference thermometers every week to avoid any false readings due to possible damage to the thermometers during data collection.

### Data processing and analysis

We discussed maintaining participant confidentiality throughout the entire data collection procedure during the training. After data was collected all the necessary information, data was processed, edited, coded, classified, cleaned, and entered into a computer. To ensure the consistency and thoroughness of the surveys, the entire process was carefully observed. The data were originally cleaned manually, coded, and entered into Epi Data version 4.6 before being exported to SPSS (Statistical Package for Social Sciences, version 26) for analysis. After coding, and entering the data into the software descriptive analysis was done to determine the means, standard deviations frequencies, and proportions, and the study population was described about relevant factors and then presented in tables.

Bivariable analysis was used to discover candidate variables (p < 0.25) for multivariate analysis after assumptions, including dichotomous variables, multicollinearity issues, the chi-square test, and mutual exclusivity, were first validated. To control for confounders, multivariable logistic regression was used to further analyze variables that had a p-value of less than 0.25 in the bivariable analysis. As a measure of association, the odds ratio (OR) with a 95% confidence interval (CI) was utilized, and factors with a p-value less than 0.05 in the multivariable logistic regression were regarded as significantly associated variables. The goodness of fit was tested by the Hosmer and Lemeshow test, which yielded a sig = 0.95. The variance inflation factor (VIF), which should be less than 10, was used to assess the multiple collinearities. A histogram and Q‒Q plot test were used to determine the normality of the data.

## Results

### Sociodemographic characteristics of the respondents

A total of 682 mothers with neonates (49.3% from rural areas and 50.7% from urban areas) participated in the study, for a 95.25% response rate. The majority of the participants (47.3% from rural areas and 48.8% from urban areas) were aged > 20–30 years, with a mean age of 26 years. 75, and 25.43, respectively. 91% of the rural participants and 86% of the urban participants were married, whereas 65% of the rural participants and 50% of the urban participants were Protestant religious followers. Approximately 50.4% of women in rural areas and 32.7% of those in urban areas were housewives according to their occupations (Table 1).

**Table 1:** Sociodemographic characteristics of neonate’s parents in Shebadino wereda, Sidama, Ethiopia 20,222

Variables	Category	Rural (*n* = 336)		Urban (*n* = 346)	
		**Frequency**	**Percent**	**Frequency**	**Percent**
Mother’s Age	< 20	121	36	115	48.8
	20–30	159	47.3	169	48.8
	> 30	56	16.7	62	17.9
Marital Status	Married	304	91	298	86
	Divorced	14	4	12	4
	Single	18	5	36	10
Religion	Protestant	218	65	174	50
	Orthodox	111	33	162	47
	Muslim	7	2	10	3
Occupation	Farmer	121	36	35	10.1
	Housewife	171	50.4	113	32.7
	Governmental employ	19	5.6	87	25.1
	Private business	25	7.4	111	32.1
Husbands Occupation (*n* = 304 for rural and 298 for Urban	Farmer	216	71	57	19
	Private business	72	24	145	49
	Government employ	16	5	96	32
Educational status	Unable to read & write	138	41	112	32.4
	Read & write	89	26.5	103	29.7
	Elementary school	50	15	75	21.7
	High school/preparatory	42	12.5	25	7.2
	Above grade 12	17	5	31	9
Mother has her income	Yes	158	47	199	57.5
	No	178	53	147	42.5
Wealth index	Richest	7	2	18	5
	Richer	28	8	41	12
	Middle	141	42	163	47
	Poorer	93	28	79	23
	Poorest	67	20	45	13

### Obstetric characteristics of the mothers

Two hundred forty (71.4%) of the mothers from rural areas and three hundred thirty-three (96.2%) of the mothers from urban areas had visited health facilities for antenatal care (ANC) during their recent pregnancy at least once. Overall, 175 (52.1%) of the mothers from rural areas and 53 (15.3%) of the mothers from urban areas were given birth at home. A total of 166 (77%) infants from rural areas and 185 (75%) from urban areas were delivered at >  = 37 completed weeks. Approximately 268 (79.4%) of the neonates from rural areas and 272 (78.6%) from urban areas were delivered alone (Table 2).

**Table 2:** Obstetric characteristics of the neonate mothers in Shebadino Woreda, Sidama region, Ethiopia, 2022 (*n* = 682)

Variables	Category	Rural		Urban	
		**Frequency**	**Percent**	**Frequency**	**Percent**
Gestational age in weeks (n1 = 216 and n2 = 248)	< 37 weeks	50	23	63	25.4
	> = 37 weeks	166	77	185	75
ANC follow-up during the last pregnancy	Yes	240	71.4	333	96.2
	No	96	28.6	13	3.8
Number of ANC visits (n1 = 240, and n2 = 333)	< 4	88	37	146	44
	> = 4	152	63	187	56
Obstetric problem during the last pregnancy/labor(n = 488)	Yes	112	33.3	70	20.2
	No	224	66.7	276	79.8
Place of birth	Health facility	161	47.9	293	84.7
	Home	175	52.1	53	15.3
Onset of labor	Spontaneous	240	71.4	237	68.5
	Cesarean section	53	15.8	66	19.1
	Induced	43	12.8	43	12.4
Birth attendant	Health provider	161	47.9	298	86.1
	Traditional birth Attendant	60	17.9	22	6.4
	Family	115	34.2	26	7.5
Parity	1–3	169	50.3	166	48
	4–6	127	37.8	136	39.3
	> 6	40	11.9	44	12.7
Type of delivery	Cesarean birth	53	15.8	66	19
	Spontaneous Vaginal births	283	84.2	280	81
Number of the child delivered	Single	268	79.8	272	78.6
	Twin or more	68	20.2	74	21.4

### Behavioral and neonatal factors

One hundred seventy-one (50.9%) were from rural areas, and one hundred eleven (32.7%) of the neonates from urban areas were bathed within 24 h after delivery. A majority (142 [42.3%] of the neonates were from rural areas, and 234 [67.6%] were from urban areas; these neonates were placed on the mothers’ abdomens (skin-to-skin contact) after delivery. Traditional medication (Amessa) was used for 195 (58%) neonates from rural areas and 204 (60.7%) neonates from urban areas. Among the total participants, 197 (57.7%) were rural, and 217 (62.7%) were older than 7 days, with mean ages of 11.21 and 9.35 days, respectively **(**Table 3**).**

**Table 3:** Behavioral and neonatal factors among neonates in Shebadino Woreda, Sidama, southern Ethiopia 2022 (*n* = 682)

Variables	Category	Rural		Urban	
		**Frequency**	**Percent**	**Frequency**	**Percent**
Baby bathed within 24 h Water	Yes	171	50.9	111	32.7
	No	165	49.1	235	67.9
Water used to bath the baby (n1 = 171, n n2 = 111)	Warm	97	56.7	65	58
	Cold	74	43.3	46	41
Baby breastfed within one hour	Yes	241	71.7	306	88.4
	No	95	28.3	40	11.6
where baby placed after delivery (Skin to skin contact))	On mother’s abdomen	142	42.3	234	67.6
	Covered with Cloth	138	41	61	17.6
	I don’t know	56	16.7	51	14.8
Head covered with cap	Yes	240	71.4	269	77.7
	No	96	28.6	77	22.3
Baby wrapped with dry clothing after bathing	Yes	282	84	248	71.7
	No	54	16	98	28.3
Baby kept apart from mother	Yes	87	26	127	36.7
	No	249	74	219	63.3
Any traditional practice done	Yes	195	58	204	60.7
	No	141	42	132	39.3
Baby took food by mouth	Yes	186	55.4	199	57.5
	No	150	44.6	147	42.5
Food taken by mouth(n1 = 86, n2 = 199)	Amessa	120	64.5	119	59.8
	Water	39	21	47	13.6
	Milk	27	14.5	33	16.6
Neonatal weight	≥ 2500 gm	318	94.6	58	16.7
	< 2500 gm	18	5.4	288	83.3
Sex	Male	161	47.9	159	46
	Female	175	52.1	187	54
Age of neonate	≥ 7	194	57.7	217	62.7
	< 7	142	42.2	129	37.3

### Environmental factors

The majority, one hundred ninety-five (58%) rural and two hundred fifteen (62.1%) urban neonates, were delivered at night. Approximately 176 (52.4%) of the rural neonates' mothers and 137 (40.2%) of the urban neonate families placed cold objects or metal near the bed of the baby, whereas 131 (39%) and 217 (62.7%) of the rural and urban neonate mothers, respectively, were non-hypothermic** (**Table 4**).**

**Table 4:** Environmental conditions assessed during the time of data collection from neonates in the shebadino woreda, Sidama region, southern Ethiopia, 2022 (*n* = 682)

variables	Category	Rural		Urban	
		**Frequency**	**Percent**	**Frequency**	**Percent**
Time of delivery	Day	141	50.3	131	37.9
	Night	195	58	215	62.1
The room was warmed, before and after delivery	Yes	169	50.3	230	66.5
	No	167	49.7	116	33.5
Cold objects or metal near the bed of the baby	Yes	176	52.4	139	40.2
	No	160	47.6	207	59.8
Human and animal houses separated	Yes	174	51.8	332	96
	No	162	48.8	14	4
Room temperature	< 20	182	54	106	30.6
	> = 20	154	46	240	69.4
Mother body Temperature	< 36.5	205	61	129	37.3
	> = 36.5	131	39	217	62.7

### Prevalence of neonatal hypothermia

The overall prevalence of neonatal hypothermia in this study was 51.8% (95% CI: 47.2%-56.3%). The prevalence of neonatal hypothermia was higher among rural neonates, 55.1% when compared to those of urban neonates 48.6%. The mean axillary temperature was 36.25 °C (SD ± 1.24) and 36.54 °C (SD ± 1.51) for rural and urban areas, respectively. Overall, 22.6% (95% CI 20.9 to 26.4) have mild hypothermia (temperature 36.0 °C to < 36.5 °C), whereas 29.2% (95% CI 25.8 to 34.4) have moderate hypothermia (temperature 32.0 °C to < 36.0 °C). No neonate was recorded as severe hypothermia (temperature less than 32.0 °C).

### Factors associated with neonatal hypothermia

#### Factors associated with neonatal hypothermia among neonates in rural Kebeles

Factors that were significantly associated with neonatal hypothermia among neonates from rural areas according to the bivariable analysis were not warming room, no history of ANC follow-up, age of neonate, giving baby food by mouth, applying traditional medicine to neonates, breathing babies before 24 h, putting cold objects near babies’ beds, not separating animals and humans and maintaining mothers’ body temperature.

According to the multivariate logistic regression, four variables were found to be significantly associated at a p-value of < 0.05. Those neonates who consumed traditional medication by mouth were 1.83 times more likely to be hypothermic than those who were on exclusive breastmilk (AOR = 1.83, 95% CI: 1.45–3.24). Additionally, neonates who slept near metal or other cold objects were 2.64 times more likely to be hypothermic than those who were not (AOR = 2.97, 95% CI = 1.75–5.03). Those neonates who slept in rooms where human and animal houses did not separate were 2.3 times more likely to develop hypothermia than those whose houses were separated (AOR = 2.3, 95% CI: 1.67–3.52). Neonates who were bathed within 24 h were 3.64 times more likely to develop hypothermia when compared to their counterparts (AOR = 3.64, 95% CI:1.39–7.16) Table 5.

**Table 5:** Bivariable and multivariable logistic regression models with cross-tabulation for factors associated with neonatal hypothermia among rural neonates in the Shebadino woreda, Sidama Region, southern Ethiopia, 2022 (*n* = 336)

Variables	Response	Hypothermic (185)	Non-Hypothermic (151)	COR (CI 95%)	AOR (CI 95%)	*P* value
Room warmed	Yes	67	84	1	1	1
	No	103	83	1.55(1.02–4.71)	1.25(0.74–2.10)	0.41
Have ANC follow-up	Yes	97	54	1	1	1
	No	143	42	1.89(0.98–3.73)	0.56(0.29–0.98)	0.51
Age of neonate	> = 7	47	104	1	1	1
	< 7	99	86	0.392(0.25–0.65)	0.42(0.24–0.65)	0.00
Bathing within 24 h	Yes	123	44	4.82(2.21–9.50)	**3.64(1.39–7.16)**	**0.012**
	No	62	107	1	1	1
Baby has taken traditional medication by mouth	Yes	117	68	2.04(1.32–3.17)	**1.83(1.04–3.20**)	**0.0035**
	No	69	82	1	1	1
applying for traditional medicine	Yes	122	73	2.07(1.31–3.21)	1.18(0.62–2.24)	0.59
	No	63	78	1	1	1
putting cold objects near babies’ bed	Yes	111	65	1.98(1.28–3.07)	**2.97(1.75–5.03)**	0.000
	No	74	86	1	1	1
Mothers body temperature	< 36	92	59	1.54(0.99–2.38)	2.25(1.34–3.76)	1
	> = 36.5	93	92	1	1	1
animals and humans house separated	Yes	103	71	1	**1**	1
	No	82	80	1.42(0.85–4.6)	**1.75(1.05–2.91)**	0.031

#### Factors associated with neonatal hypothermia among neonates in urban kebeles

Factors such as the number of neonates delivered, time of delivery, place of a cold object near the baby, use of dry clothes, previous NICU admission history, and the use of traditional medicine for neonates were found to be significantly associated with neonatal hypothermia among neonates from urban areas according to the bivariable analysis (P value < 0.25).

According to the multivariate logistic regression, three variables were found to be significantly different at a p-value < 0.05. Among them, neonates who slept near a cold object (metal) were 2.01 times more likely to develop hypothermia than those who did not (AOR = 2.01, 95% CI: 1.59–4.38). Additionally, neonates who started taking amessa for traditional medication were 3.11 times more likely to develop hypothermia than their counterparts (AOR = 3.11, 95% CI: 1.85–5.21). At night, neonates who were delivered were 1.81 times more likely to develop hypothermia than were those who were delivered during the day (AOR = 1.81, 95% CI = 1.01–5.02) Table 6**.**

**Table 6:** Bivariate and multivariate logistic regression models with cross-tabulation for factors associated with neonatal hypothermia among urban neonates in the Shebadino region, Sidama region, southern Ethiopia, 2022 (*n* = 346)

Variables	Response	Hypothermic (168)	Non-Hypothermic (178)	COR (CI 95%)	AOR (CI 95%)	*P* value
Number of neonates delivered	Single	142	143	1	1	1
	Two or more	26	35	1.34 (0.89–4.71)	0.44(0.24–1.08)	0.07
Cold object near bead	Yes	88	56	2.40(1.37–3.29)	**2.01(1.59–4.38)**	**0.000**
	No	80	122	1	1	1
Time of delivery	Day	107	68	1	1	1
	Night	61	110	2.83(0.85–5.94)	**1.81(1.01–5.62)**	**0.004**
Baby took traditional medication by mouth	Yes	118	68	3.81(1.44–6.35)	**3.11(1.85–5.21)**	**0.000**
	No	50	110	1	1	1
Number of ANC visit(*n* = 333)	> = 4	117	88	2.63(0.97–7.2)	1.18(0.62–2.24)	0.59
	< 4	43	85	1	1	1
Baby wrapped with a dry cloth	Yes	120	42	8.09(3.53.-13.07)	4.12(2.35–9.11)	0.13
	No	48	136	1	1	1

#### Overall factors associated with neonatal hypothermia among neonates in the Shebedino Woreda Sidama region of southern Ethiopia

Babys who were previously admitted to the NICU were 3.88 times more likely to develop hypothermia than those who were not previously admitted to the NICU (AOR = 3.88, 95% CI = 1.52–8.37). Neonates, whose mothers had obstetrical complication(s) during pregnancy/labor were 2.38 times more likely to develop hypothermia when compared to their counterparts (AOR = 2.38 95% CI: 1.05–5.14**)**. Individuals who put a cold object (metal) near a baby’s bed were 4.20 times more likely to develop hypothermia than were those who put a cold object (metal) near a baby’s bed (AOR = 4.20, 95% CI: 2.24–7.71) Table 7**.**

**Table 7:** Bivariate and multivariate logistic regression models with cross-tabulation for factors associated with neonatal hypothermia among neonates in the Shebadino woreda, Sidama region, southern Ethiopia, 2022 (*n* = 682)

Variables	Response	Hypothermic (353)	Non-Hypothermic (329)	COR (CI 95%)	AOR (CI 95%)	P value
Number of neonates delivered	Single	311	229	1	1	1
	Two or more	42	100	3.23 (1.74–7.01)	0.32(0.17–0.61)	0.06
Cold object near bead	Yes	234	101	4.43(1.62–8.71)	**4.20(2.24–7.71)**	**0.000**
	No	119	228	1	1	1
Time of delivery	Day	130	142	1	1	1
	Night	223	187	0.77(0.38–1.02)	0.59(0.33–0.1.5)	0.087
applying for traditional medicine	Yes	236	158	2.18(1.60–2.95)	1.34(0.77–2.48)	0.285
	No	117	171	1	1	1
Previous NICU admission history	Yes	194	68	4.68(2.45–6.69)	**3.88(1.52–8.37**)	**0.003**
	NO	159	261	1	1	1
Have ANC follow-up	yes	304	269	1	1	1
	No	49	60	1.38(0.91–2.08)	0.57(0.34–1.03)	0.06
presence of obstetrical complication(s) during pregnancy/labor	yes	144	70	2.55(1.04–9.31)	**2.38(1.05–5.14)**	**0.001**
	No	209	259	1	1	1
The baby started taking food by mouth	Yes	230	155	2.12(1.56–2.88)	1.66(0.93–2.97)	0.087
	No	122	174	1	1	1

## Discussion

The overall prevalence of neonatal hypothermia in this study was 51.8% (95% CI: 47.2%-56.3%). The prevalence of neonatal hypothermia was 55.1% among rural neonates and 48.6% among urban neonates, which showed that there was a significant difference in the incidence of neonatal hypothermia among neonates in the shebedino woreda (*p* = 0.003). These findings revealed that there was significantly greater neonatal hypothermia in rural neonates than in urban neonates. This difference may be due to the lack of thermal care provided to rural neonates compared to urban neonates.

The overall prevalence was comparable to that reported in previous studies in the Islamic Republic of Iran (53%) [17] and in northern Uganda (51%) [18]. Another study conducted in Pakistan revealed that the prevalence of neonatal hypothermia was 49.5% [19], which is in line with our study. Additionally, these findings are in line with those of a study conducted at Arbaminch General Hospital (50.3%) [11]. This similarity may be due to the study sites and the large sample size used.

However, the overall prevalence found in our study was 45% higher than that in another community-based study conducted in India [3]. This variation might be due to seasonal conditions, data collection tools, differences in temperature measurement sites, or economic and cultural differences in those communities. Additionally, the overall prevalence of neonatal hypothermia was higher than that in another study conducted among home-delivered neonates in North India (11%) [20]. This was due to Hypothermia definition variation as the author of that research defined neonatal hypothermia as a temperature less than 35.6 °C whereas we defined it based on WHO definition recommendation definition [13]. This study is also less than the study conducted in Addis Ababa Ethiopia with a proportion of 83.17% [21].

These findings are lower than those of other studies conducted at the hospital, in which 77% of the participants were admitted to the neonatal intensive care unit in tertiary hospitals in Malawi [22], 69.8% were admitted to Gonder Teaching and References Hospital [14], and 64% were admitted to Addis Ababa Hospital [14]. The possible reason for this difference is that neonates who were admitted to the NICU had different indications, which could decrease their ability to adapt to the external environment out of the womb and easily develop hypothermia. On the other hand, unlike those studies, late neonates were included in our study; neonates can resist heat loss as they age and can easily defend against hypothermia [14] (Mulatu T: College of health sciences school of public health department of preventive medicine epidemiology and biostatistics track, forthcoming).

In rural areas, neonates who were bathed within 24 h after delivery were 3.64 times more likely to develop hypothermia than their counterparts were. This finding was supported by studies conducted in southern Ethiopia [11] and in the central zone of Tigray [23], and Uganda [24]. A possible explanation is that due to the effect of cold water and exposure of neonates to cold environments, neonates can be easily exposed to cold water, and their thermal temperature may decrease. Additionally, at the time of bathing, a neonate must be separated from the mother’s body contact, which can again increase the risk of hypothermia.

Another factor that has been associated with neonatal hypothermia among neonates in both rural and urban areas was administering amessa as a traditional medication. Those neonates who started drinking amessa for traditional medication were 1.83 and 3.11 times more likely to develop hypothermia in rural and urban areas, respectively, than were those who were exclusively breastfeeding. This medication was given as people of this woreda believed that this medication protects neonates against the eyes of the devil. However, this procedure results in hypothermia because it interferes with exclusive breastfeeding. The more the neonate breastfeeds, the more likely they are to have adequate glucose to cope with their energy expenditure. This is because breast milk is full of different vitamins and calories. Additionally, there is skin-to-skin contact during breastfeeding, and neonates can share maternal body heat and take advantage of their counterparts [14, 25]. There was no previous study finding with the same variables.

Sleeping near cold objects (metals) was significantly associated with hypothermia among neonates in rural and urban areas; overall, in this study, hypothermia was associated with hypothermia in 1.75, 2.01, and 4.2 times more likely to be related to their counterparts. The object was placed at the head of the bed near the baby's head. This difference in metal use may be due to people's spiritual beliefs and lack of awareness of environmental thermal care in rural communities. In contrast, neonates lose heat through conduction (neonate body contact with cold objects) and radiation (loss of heat to the cold metal surface even if not in contact). By this mechanism, neonates may lose internal heat, which can result in hypothermia [1, 13]. As no other study has been performed on this topic, it was difficult to compare our results with the other ones.

Neonates who slept in a house where humans and animals were not separated were 2.3 times more likely to develop hypothermia than those whose houses were separated from neonates in rural areas. This may be because the animals were in a wet environment with high humidity, which resulted in a decreased temperature. This may be due to heat loss in cold environments caused by radiation from the infant to a cooler environment [1]. Additionally, these neonates are easily exposed to neonatal disease, which again results in neonatal hypothermia. This variable is also a new finding, and we cannot discuss it further.

The current study revealed a significant association between the time of delivery and neonatal hypothermia among neonates in urban areas. This may be attributed to the temperature difference at night and during the day. Additionally, there is no added heat during cold nights, and newborns are at risk of losing heat and developing hypothermia [1, 21]. On the other hand, work overload during the nighttime is not equal to that during the daytime for neonates who were delivered at health institutions. These findings are in line with those of another study conducted at a Public Hospital in Addis Ababa [14], Dessie Referral Hospital [27], Northwest Ethiopia [14]. A possible explanation for this difference may be that 60.1% of the newborns in our study were delivered at night, which is similar to what was observed in the above study. For example, in the Dessie referral hospital [26] and Northwest Ethiopia, 69.8% of the deliveries were at night [14].

The overall study revealed that neonates who underwent resuscitation at birth were 3.88 times more likely to be hypothermic than those who did not. This may be because neonates who need resuscitation are those who have birth asphyxia. For those neonates, there is not enough oxygen needed for mitochondrial oxidation in brown adipose tissue for heat production. Additionally, during resuscitation, thermal care may not be provided properly without wrapping the baby on the cold table. This finding is supported by a study conducted at Gonder University Teaching Hospital [14] and Dessie Reference Hospital [26]; a study performed in Bangladesh [27]; and a study performed in Iran [28].

Another significant factor in the overall analysis was obstetrical complications, such as premature rupture of membranes, hypertension, DM, and antepartum hemorrhage during pregnancy and/or labor. Those women need special management, such as cesarean section or instrumental deliveries. Therefore, mothers might be too sick for their neonates to be in close contact during delivery, especially for skin-to-skin contact. Additionally, neonates who are born after premature rupture of the membrane might develop neonatal sepsis and a decrease in body temperature. This finding is supported by research conducted in southern [11] and eastern Ethiopia [16]. This similarity may be due to the socioeconomic status of society across the country, which leads to obstetric complications and may ultimately lead to neonatal hypothermia.

This study has many implications as it found the variation of prevalence and identified the regional variations in the severity of neonatal hypothermia between rural and urban areas. Hopefully, this information can help with healthcare planning and resource allocation, allowing policymakers to target interventions and allocate resources based on the specific needs of each setting. Additionally, we gained a deeper understanding of the factors contributing to neonatal hypothermia in different contexts. Understanding these differences can guide the development of targeted interventions to address specific factors in each setting which is very important for both the governmental and non-governmental organizations in the study region.

## Limitations and strengths of the study

### Strength of the study

With our maximum search engine, this is the first purely community-based assessment of neonatal hypothermia in Ethiopia and the second in sub-Saharan Africa after a study conducted in Northern Uganda in 2021 [18]. We found new variables that were not similar to those previously reported at the hospital level. Additionally, the findings obtained are generalizable to all neonates in the woreda, including home births.

### Limitations of the study

Compared with mercury thermometers, digital thermometers might slightly overestimate or underestimate temperature readings. We have used it because, unlike mercury thermometers, it is easily available and good for field studies [29]. The temperature was measured only twice at the same time. The instrument used by the person who performed the measurement, the site, and the duration of measurement might not be similar for all neonates, which may bias the results. Our study was performed in one season, and considerations such as seasonal variations were not taken into account. On the other hand, hospital-related characteristics, such as the qualifications of healthcare personnel working in delivery rooms and NICUs, were not taken into consideration because they may have been related to our dependent variable. Another limitation of our study was recalling bias. To decrease this possibility, proper definition and articulation of the research questions were provided, and the interviews were administered properly and consistently. The outcome of the neonates, including those referred to health institutions, was unknown. Another limitation of the study was social desirability bias.

## Conclusion and recommendations

### Conclusion

The overall prevalence of neonatal hypothermia in this study was 51.8% (95% CI: 47.2%-56.3%). The prevalence of neonatal hypothermia was greater among rural neonates (55.1%) than among urban neonates (48.6%). Health extension workers should work on Community-based neonatal care and teach society to not practice traditional medications, separating humans and animals' houses and putting cold metal on the bed under the neonatal head.

### Recommendations

Based on our study findings, the following public health measures were recommended for woreda health sector management. It is better to provide periodic training for HEWs for cost-effective thermal care such as warming the room, separating animals' houses and animals, and teaching on cultural malpractice which affects neonatal body temperature. This approach involves ensuring good awareness, knowledge, and skills of HEWs to implement prevention mechanisms such as room warming, neonatal wrapping (head covering), continuing skin–skin contact, separating humans and animal houses, and separating a cold object from a neonate's bed. Again, society's teaching on the effect of traditional practices performed on neonates is needed.

### For public health institutions

Proper counseling on the effect of traditional practice and proper thermal care should be provided before discharge to home, especially for those who come from rural society. Nighttime delivery room manpower should be prioritized. Additionally, counseling for cost-effective thermal care, such as in a warm environment where neonates can sleep, and counseling to give birth at health institutions during ANC follow-up is also mandatory Additionally, counseling on birth preparedness during ANC for neonates covering material should also be mandatory.

### NGOs working in this area

It would be good if NGOs working in this area were alert and run for solutions to prevent hypothermia in the woreda based on our study findings and to address these gaps.

### Supplementary Information

Supplementary Material 1.

## Data Availability

The datasets used or analyzed during the current study are not publicly available. We did not have consent from all participants to publish the raw data but are available from the corresponding author upon reasonable request.

## Abbreviations

AOR: Adjusted odds ratio
CI: Confidence interval
CBNC: Community-based neonatal care
CFR: Case fatality rate
COR: Crude odds ratio
EDHS: Ethiopia demographic health survey
EMDHS: Ethiopia Mini demographic health survey
ENC: Essential newborn care
HEW: Health extension worker
IRB: Institutional Review Board
KMC: Kangaroo Mother Care
MDG: Millennium Development Goals
WHO: World Health Organization
