# Supplementary material for: Comparative study of neonatal hypothermia and associated factors among neonates in rural and urban areas of the Shebadino Woreda, Sidama region, Southern Ethiopia: a community-based comparative cross-sectional study
Source: BMC Public Health. 2024 Jul 20;24:1945. doi: 10.1186/s12889-024-19504-8 (PMC11264896; doi:10.1186/s12889-024-19504-8)
Supplement: Supplementary file 1 — Supplementary Material 1. [file 12889_2024_19504_MOESM1_ESM.docx]

ENGLISH VERSION OF INFORMATION SHEET

**Principal investigator**: Gizu Tola

**Title of the research**: **Comparative study of neonatal hypothermia and associated factors among neonates in rural and urban areas of the Shebadino woreda, Sidama region, southern Ethiopia: a community-based comparative cross-sectional study**

Greetings; Good morning / afternoon my name _______________. I am here on the behalf of Gizu Tola, a postgraduate student from Hawassa University, currently carrying out research to determine Neonatal Hypothermia and factors associated with it among neonates in Shebadino woreda, Sidama Region South Ethiopia. Unfortunately, you are chosen to participate in this study. Before you decide whether to participate in this study, I would like to explain to you the objective of the study, any risks, benefits, procedure and what is expected from you.

**Purpose of the Research Project**: to determine Neonatal Hypothermia and factors associated with it among neonates in Shebadino woreda, Sidama Region South Ethiopia.

**Risk and/or Discomfort**: -The study will be conducted by taking appropriate information from the study participant; it will not cause any problem on the client. The name or any other identifying information will not be recorded on the questionnaire and all information taken from you will be kept strictly confidential. The information gotten will be used only for the study purpose.

**Benefits**:- the research have no direct benefit for one who included in this research. However, you may be indirectly beneficial. This is because if program planners are preparing predicted plan there is a benefit for clients in the program of getting appropriate care and treatment services.

**Confidentiality:** To reassure confidentiality the data will be collected by those individuals who are health professionals and the information will be collected without the name of the clients. The information collected from this research project will be kept confidential. In addition, it will not be revealed to anyone except the principal investigator.

INFORMED CONSENT

I have been explained all information and procedures that are part of this research study and I have understood. I understand that the research imposes no risk on my life and therefore no compensation would be provided. I hereby agree to participate in this research study and give my voluntary consent. I hereby also give rights to the researcher for collecting the data that are required for the study.

Agree Disagree

Name of the Interviewer____________________ Signature__________ Date ___________

Name of the Supervisor_____________________ Signature__________ Date ___________

Researcher: Gizu Tola

Phone No: +251 9 32499835

Email: gizutola2020@gmail.com

Code No._____________ woreda ____________Kebele____________

Part-I socio-demographic characteristics of the mother

| SN | Question | Response | Remark |
| --- | --- | --- | --- |
| 101 | Age of the mother | ……………. Years |  |
| 102 | What is your occupation? | 1. Housewife  2. Government employee  3. Private business  4. Farmer  5. Other (Specify |  |
| 103 | What is your marital status? | 1. Married  2. Divorced  3. Single  4. Widowed  5. Separated | Go to Q # 6 if not married |
| 104 | What is the occupation of your husband | 1.Government employee  2.Private business  3.Farmer  4.Other (Specify)__________ |  |
| 105 | To which religious groups do you belong? | 1.Orthodox  2.Protestant  3.Muslim  4.Other (Specify)______________  ________________________ |  |
| 106 | What is the highest grade you completed? | 1.Unable to read & write  2.Read and write  3.Elementary school (grade 1 -4)  4.Secondary school(Grade 5-8)  5.High school/prep.(grade 9 -12)  6.Above grade 12 |  |
| 107 | What is the educational level of your husband? | 1.Unable to read & write  2.Read and write  3.Elementary school (grade 1 -4)  4.Secondary school(Grade 5-8)  5.High school/prep.(grade 9 -12)  6.Above grade 12 |  |
| 108 | Does the house has electrify | 1. Yes  2.No |  |
| 109 | Do you have your own income? | 1. Yes  2.No | If not go to Q#12 |
| 110 | What is your monthly income level in ETB | __________ ETB |  |
| 111 | What is the monthly income level of your husband? | ___________ ETB | Write NA if not married |
| 112 | Where are you living now? (Residence) | 1. Urban area  2. Rural area |  |
| 113 | How far is your home from the health facility? | 1) <10km  2) >10km |  |

Part II Obstetric Characteristics

| SN | Question | Response | Remark |
| --- | --- | --- | --- |
| 201 | How many times did you gave birth? (Parity) | _______________ times |  |
| 202 | Do you have an ANC follow-up during the most recent pregnancy? | 1. Yes  2.No | Go to Q # 9 if NO |
| 203 | If yes, how many times did you visit the ANC clinic | ________ times |  |
| 204 | Have you ever been told by your ANC provider that there was a problem with your pregnancy? | 1. Yes  2.No |  |
| 205 | If yes, what was the problem | 1. Hypertension  2. Bleeding  3. DM  4. PROM  5.Other (Specify)_______ |  |
| 206 | Can you tell me the total duration of your labor in hours? | _______________ hours | NA if elective C/s |
| 207 | How was your labor started? | 1. Spontaneous  2.Initiated with medication. | NA if elective C/S |

Part III Neonate related questions

Physiological Factor

| SN | Question | Response | Remark |
| --- | --- | --- | --- |
| 301 | Sex of the neonate | 1. Male  2. Female |  |
| 302 | Current Birth Weight in grams | ____gms |  |
| 303 | Gestational age at delivery in weeks | ____ |  |
| 304 | Age of the neonate after delivery in days | _____ hrs/days . |  |
| 305 | Neonate Axillary Temperature in degree Celsius | ____ |  |
| 306 | Number of delivered neonate | 1.Single  2.Twin  3. triple, and above |  |

Behavioral factors

| SN | Question | Response | Remark |
| --- | --- | --- | --- |
| 401 | Place of delivery | 1.Home  2.Health facility |  |
| 402 | Who assisted you during the birth of this baby? | 1. Family member /relative  2. Traditional birth attendant  3. Health extension worker  4. Health professional  5.No one (by myself) |  |
| 403 | Type of delivery | 1.SVD  2.C/S  3).instrumental delivery | If, instrumental delivery, ask the mother what does the instrument looks like |
| 404 | Was the baby bathed within 24hours after delivery? | 1. Yes  2.No | If No, go to the next Q no 6 |
| 405 | Type of water used to bath the baby | 1. Warm water  2. Coldwater |  |
| 406 | After finishing washing the baby, did the caregiver dress the neonates immediately? | 1. Yes  2.No |  |
| 407 | Was the baby breastfed within 1 hour after delivery? | 1. Yes  2.No | If No, go to the next Q |
| 408 | If yes,was colostrum extracted? | 1. Yes  2.No |  |
| 408 | was the baby put On the mother's abdomen  immediately after delivery? | 1. Yes  2.No |  |
| 409 | Was the baby’s head covered after delivery with a cap? | 1. Yes  2.No |  |
| 410 | Was the baby wearing Socks after delivery? | 1. Yes  2.No |  |
| 411 | Was the baby wearing a dry clean cloth after drying thoroughly? | 1. Yes  2.No |  |
| 412 | Was the baby separated from you, and sleeping in another room after delivery? | 1. Yes  2.No |  |
| 413 | If yes, what was the reason and where did the neonate stay? | __________________  __________________ |  |
| 414 | Has a health provider told you that your baby faced difficulty breathing at hospital? | 1. Yes  2.No |  |
| 415 | Does amessa given to neonate? | 1. Yes  2.No |  |
| 416 | Was the neonate given food by mouth yet? | 1. Yes  2.No |  |
| 417 | If yes, what? | 1. Water  2. Better  3. Oil  4. Milk  5.Other (specify)______ |  |
| 418 | Did the neonate manifested another symptom | 1. Yes  2.No | If No Go to the next Q |
| 419 | If yes, what are the sign and symptoms the baby manifested? | 1) Apnea  2) Chest in-drawing  3) Fast breathing  4) Abdominal distension  5) Not moving well  6) Fever  7) Vomiting | More than one answer is possible |
| 420 | Pulse rate | ____ |  |
| 421 | Oxygen Saturation | ____ |  |
| 422 | Does the neonate have congenital malformation? | 1. Yes  2.No |  |
| 423 | If yes, mention it. |  | __________________  __________________ |

Environmental Factors

| SN | Question | Response | Remarks |
| --- | --- | --- | --- |
| 501 | At what time of the day, is a baby born? | 1. Nighttime  2. Daytime |  |
| 502 | Does the room warm, before and after delivery? | 1 Yes  2 No |  |
| 503 | Is there a cold object or metal nearby  the bed of the baby? | 1 Yes  2 No |  |
| 504 | Is there an extremely hot item nearby the baby's bed or place where the baby is slipping? | 1 Yes  2 No |  |
| 505 | Does a room have a window? | 1 Yes  2 No |  |
| 506 | If yes, How many of them are a function | ______ |  |
| 507 | Does a human's and animal's house separate? | 1 Yes  2 No |  |
| 508 | What is the current room temperature in degrees Celsius? | 1) >=20°C  2) < 20°C |  |
| 509 | Mother's body temperature in degrees Celsius? | ______ |  |
| 510 | How was the baby traveled home after delivery? | 1 carried by family  2 public transportation | Skip if delivered at the hospital |

**ANNEX 3:**

SIDAAMU AFII XAMOTE WORAQATA

**Gaffa mite: Sidaamu afii mashalaqetena qoola**

**Xiinxalo assanohu: Gizu tola**

**Xiinxalote umo**:Marqu qaaquli qiijajishate xibina amadisiisantino korkaatuba Sidaamu qoqowi shabadiino woradira gido 2022 .

**Keere:** keere galtini/hosini sum'ya_______________ yinanie. Ani hawassi universte layinki digre rosaancho ikinohu Gizu Tola yinanihu, Marqu qaaquli qiijajishate xibina amadisiisantino korkaatuba Sidaamu qoqowi Shabadiino woradira gido yaano umini loosani afamanohu widooti. Tene xiinxalo gido beeqate ati dooramota. Ikolalana beeqte albaani, ani atera xiinxalote umo, mixo, jado,horona atewini agaramanore xawise kuleemohe.

**Xiinxalote hasato**: 2022 Marqu qaaquli qiijajishate xibina amadisiisantino korkaatuba Sidaamu qoqowi Shabadiino Woradira gido bade afate.

**Dano/gawajona horo:** xiinxalo mitu manchi/mancho aana gawajo diabitano. Ani mito mitonka mana, balchoomana dagana dagooma lowontani ayiiriseemo. Hatono tini xiinxalo tumo/dawaro aano manira/ amuwira horontani baatoshu dinose. Ikolana xiinxalo tini wole doogoni dawartu manira iko wolu manira horo uyiitano yee hedeemo.

**Agarooshe:**su'maki tumo/dawaro qolata woyiite diboreesinani. Mitunku tumo/dawaro ayiirano diuyiinani/diodeesinani. Hatono dawara hasirataki xa'mora dawara hooga dandaata.tene xiinxalo darga gantanohu ate dawaroniiti. Hako daafo asootoe/ae/ootae dawaro/tumo daafira lowontani galaxeemo'he

**ANNEX 4:**

**SUMUUMMETE WORAQATA**

Tene mashalaqebaala iillitinoenna heeltinoe. Tine xiinxallo aana’ya illishanno gawajjo nookkita afoomnto daafira baantannie batooshi dino. Umo’ya fajje harunsate sumuume’ya xawiseemmo. Qohe kae xinxalaanchoho hasiisannosi mashalaqe afiranno gede wo’ma fajjo eemosi

Summu yeemma______________ summu diyeemma ______________

Xa’mamaanchu su’ma ______________ malaate ______________ Barra______________

Qorqoraanchu su’ma malaate ______________ malate______________ Barra ______________

Xiinxalaanchu Gizuu Tolaa

Bilbilu kiiro 09 32499835

‘’Emeelle’’gizutola2020@gmail.com

Baxxitino kiiro ______________ Worada ______________ Qawale_____________

Sidamo Affo

Demographic characteristics of the mother

| SN | Xa’mo | Dawaro | Remark |
| --- | --- | --- | --- |
| 101 | 2. Sai diro dirikki me'eho? | __________Diro |  |
| 102 | 3. Loosikki maa'ti? | 1. Mini ama  2. Mangistete Looso  3. U’miya Looso  4. Baatto loosire Galeema  5. Saadate ce’o  6. Wole looso(Xawisi)________ |  |
| 104 | 4. Adhamate gari maa lawanno? | 1. Mine asiroomate  2. Tiroomate  3. Mine diassiroomate  4. Gunnitete  5. Tiroomate | Mine asirootakkiro xa’mote kiiro 6 lai |
| 105 | 5. Minaanikki loosu dani maati? | 1. Mangistete Looso  2. U’misi Looso  3.Baatto loosire Galanno  4. Saadate ce’o  5. Wole looso(Xawisi)________ |  |
| 106 | 6. Garekki maati? | ________________________ |  |
| 107 | 7. Amanakki maati? | 1. Ortodokise  2. Kiristiyana  3. Islaama  4.Wole amana(Xawisi)________ |  |
| 108 | 8. Rosu deerikki mageeshiho? | 1.Nabbawana boreessa didandeema  2. Nabbawana boreessa dandeema  3. Umi dirimi roso(1-4)  4.Layinkki dirimi roso(5-8)  5. Aliidi roso/qixaawote roso(9-12)  6. 12 kifile ale |  |
| 109 | Minaanikki rosu deeri mageeshiho? | 1.Nabbawana boreessa didandaanno  2.Nabbawana boreessa dandaanno  3. Umi dirimi roso(1-4)  4. Layinkki dirimi roso(5-8)  5. Aliidi roso/qixaawote roso(9-12)  6.12 kifile ale |  |
| 110 | Ate umikki e’o noohe? | 1. Ee  2. Dino’e | Dawarokki dinoe ikkituro xa’mote kiiro 12 la’i |
| 111 | Aganu e’okki kirooteni mageeshite? | ____________Birra |  |
| 112 | Minaanikki aganu e’o mageeshite? | _____________Birra | Mine assirootakkiro Dawaro dinoe yite boreessi |
| 113 | Xa mama heeratta? | 1. Katamaho  2.Baadiye |  |
| 114 | -Minikki Fayimmate uurinshawiini mageesha fafanno? | 1) 10km woro  2) 10km ale |  |

Part II Obstetric Characteristics

| SN | Xa’mo | Dawaro | Remark |
| --- | --- | --- | --- |
| 201 | Me’e higge godowootta? | _________hige |  |
| 202 | Me’e higge ilootta? | _________hige |  |
| 203 | Xaa godowira harunso asiroota? | 1.Ee  2.Dee’ni | Dawarokki dee’ni ikkituro, xa’mote kiiro 5 lai |
| 204 | Dawarokki ee ikkituro, me’e higge? | _________hige |  |
| 205 | Xaa godowira qarru no yine ku’looniheni? | 1. Ee  2. Diku’loonie |  |
| 206 | Dawarokki ee ikkituro, maati kuli’he qarri? | 1. Mundeete xiwo  2. Mundeete du’nama  3. Sukaare  4. Yanniweelo game  5Wole qarra(Xawisi)______ |  |
| 207 | -Godowu gamekki yana kula dandaata? | ________saate | Dawaro dinoe, mannu kaa’loni ilootaha ikkiro(godowa dareena ilootaha ikkiro) |
| 208 | Godowu game umiseni hanafinonso mannu kaa’lonni? | 1.Umise yannani  2.Mannu kaa’lonni  3.Diafoomma | Dawaro dinoe, mannu kaa’loni ilootaha ikkiro(godowa dareena ilootaha ikkiro) |

Part III Qaaqu ledo Amadanitno Xa’mo

Physiological Factor

| SN | Xa’mo | Dawaro | Remark |
| --- | --- | --- | --- |
| 301 | Marqu Qaaqi koo/tee | 1. koo  2. tee |  |
| 302 | Ilami woyite qelphephosi me'ete? | _________girame |  |
| 303 | Godowu gido mageesha keeshino | 1.__________Barra  2. Diafooma |  |
| 304 | Kuni Qaaqi ilaminkunni me’e seate ikkinosi? | _____ Saate. |  |

Akattu ikkito

| SN | Xa’mo | Dawaro | Hedo |
| --- | --- | --- | --- |
| 401 | - Mama ilamino? | 1.mine  2. Hospitaalete |  |
| 402 | Ilitta woyite ayeti kaa’linohehu? | 1. Mini maate/fiixu  2. Budu ogeete  3.Fayimate ogeete  4. Keeranchimate ogeeye  5.Kaa’linoehu dino(calla’ya) |  |
| 403 | Hitoonni ilootta? | 1.Gamete niixxe ilootta  2.Godowa dareena iloota  3. Siwiilu uduni iloota  4. Laasitikete uduni iloota | Uduunichu kaal’onni iltinoro, uduunichu dana xa’mi |
| 404 | Marqu Qaaqi ilamihu gedensaanni lemiina shoole saate giddo hayishinoonni? | 1. Ee  2. Dihayishinooni |  |
| 405 | Wayi hiitooho hayishinihu | 1. iibadu wayini  2. Qidadu wayini  3. Wolurini |  |
| 406 | Marqu Qaqi ilamihuni mitte saate gedensaanni unuuna Qanino? | 1. Ee  2. Diqanino | Dawarokki diqanino ikkituro, aantino xa’mo lai |
| 407 | Unuununi umi ado hunoonni? | 1. Ee  2. Dihunoonni |  |
| 408 | Marqu Qaaqi ilamihu gedensaanni amate godowi ana woronisi? | 1. Ee  2. Diworoonni. |  |
| 409 | Marqu Qaaqi ilamihu gedensaanni umu diphano woronisi? | 1. Ee  2. Diworooni |  |
| 410 | Marqu Qaaqi ilamihu gedensaanni lekate diphano Woroonisi? | 1. Ee  2. Diworooni |  |
| 412 | Marqu Qaaqi ilamihu gedensaanni moola uddano udisiinsonisi? | 1. Ee  2. Diudisiinsoonni |  |
| 413 | Marqu Qaaqi ilamihu gedensaanni atewiini tonaa onte daqiiqi ale baxxee keeshino? | 1. Ee  2. Dibaxxino |  |
| 414 | Marqu Qaaqi ilami gedensaanni baxitino kaa’lo uyinasira addi kifile eesinonisini? | 1. Ee 2. Dieesinoonni | Mine ilaminoha ikkiro aantino xa’mowa sai |
| 415 | Olaaho addi kaa’lo hasidhanno qaaqullira uyinani kaa’lo no buddu ogeeyenni? | 1. Ee  2. Dino |  |
| 416 | Dawarokki ee iikituro, hiitto kaa’looti uyinanitti? | __________________  __________________ |  |
| 417 | Marqu Qaaqi ilaminku ofooho uyinoonisi no? | 1. Ee  2. Dino |  |
| 418 | Dawarokki ee ikkituro, maati uyinoonnisiri? | 1. Waa  2. Buuro  3. Zayite  4. Ado  5. Wolere(Xawisi)______ |  |
| 419 | Is other clinical manifestation manifested? | 1. Yes  2.No |  |
| 420 | If yes what are the sign and symptoms the baby manifested? | 1) Apnea  2) Chest in-drawing  3)Fast breathing  4)Abdominal distension  5) Not moving well  6) Fever  7) Vomiting | More than one answer is possible |
| 421 | Pulse rate | _______________ |  |
| 422 | Oxygen Saturation | _______________ |  |

Qooxxeessu ikkito

| SN | Xa’mo | Dawaro | Remarks |
| --- | --- | --- | --- |
| 501 | Marqu Qaaqi ma yanna ilamino? | 1. Hasha  2. Barra |  |
| 502 | Marqu Qaaqi ilami gedensaanni minu iibbinoni? | 1. Ee  2. Di iibbino |  |
| 503 | Marqu qaaqi goxino daalasi mule qiidadu uduunichi woyi siwiilu nooni? | 1. Ee  2. Dino | Ee yiitaro aantanota dawari |
| 504 | Marqu qaaqi goxino algi mule lowo geesha iibanno uduunichi nooni? | 1.Ee  2.Dino |  |
| 505 | Minu maskoote nooho? | 1. Ee  2. Dino |  |
| 506 | Dawaro ee ikkituro, Me’e maskoote no horo uyitanno? | ___________ |  |
| 507 | Mannuna saadate mini babbaxe no? | 1. Ee  2. Dibaxino |  |
| 508 | Minu giddyi iibilli me’eho(degree Celsius)? | 1) >=20°C Celsius  2) < 20°C |  |
| 509 | Amate bisu iibbili | ______°C |  |
| 510 | Qaaqo hitoo hodhishinni massini minira? | 1. Maate hanqafe  2. Dagate hodhishinni | Hospitalete ilaminoha ikkiro. |
